# Greenhouse-Selected Resistance to Cry3Bb1-Producing Corn in Three Western Corn Rootworm Populations

**DOI:** 10.1371/journal.pone.0051055

**Published:** 2012-12-20

**Authors:** Lisa N. Meihls, Matthew L. Higdon, Mark R. Ellersieck, Bruce E. Tabashnik, Bruce E. Hibbard

**Affiliations:** 1 Plant-Insect Interactions, The Boyce Thompson Institute for Plant Research, Ithaca, New York, United States of America; 2 Plant Genetics Research Unit, Agricultural Research Service, United States Department of Agriculture, Columbia, Missouri, United States of America; 3 Department of Statistics, University of Missouri-Columbia, Columbia, Missouri, United States of America; 4 Department of Entomology, University of Arizona, Tucson, Arizona, United States of America; Universidad Nacional Autonoma de Mexico, Instituto de Biotecnologia, Mexico

## Abstract

Transgenic corn producing the *Bacillus thuringiensis* (Bt) toxin Cry3Bb1 has been useful for controlling western corn rootworm, *Diabrotica virgifera virgifera* LeConte, one of the most economically important crop pests in the United States. However, rapid evolution of resistance by this beetle to Bt corn producing Cry3Bb1 has been reported previously from the laboratory, greenhouse, and field. Here we selected in the greenhouse for resistance to Cry3Bb1 corn in three colonies of WCR derived from Kansas, Minnesota, and Wisconsin, respectively. Three generations of rearing on Cry3Bb1 corn significantly increased larval survival on Cry3Bb1 corn, resulting in similar survival in the greenhouse for selected colonies on Cry3Bb1 corn and isoline corn that does not produce Bt toxin. After four to seven generations of rearing on Cry3Bb1 corn, survival in the field on Cry3Bb1 corn relative to isoline corn more than doubled for selected colonies (72%) compared with control colonies (33%). For both selected and control colonies, survival in the field was significantly lower on Cry3Bb1 corn than on isoline corn. On isoline corn, most fitness components were similar for selected colonies and control colonies. However, fecundity was significantly lower for selected colonies than control colonies, indicating a fitness cost associated with resistance. The rapid evolution of resistance by western corn rootworm to Bt corn reported here and previously underlines the importance of effective resistance management for this pest.

## Introduction

Transgenic crops producing insecticidal proteins from the bacterium *Bacillus thuringiensis* (Bt) kill many key pests and reduce reliance on insecticide applications [1]. Bt crops covered more than 66 million hectares worldwide in 2011 [2]. Bt corn and Bt cotton targeting lepidopteran pests accounted for nearly all of the Bt crops planted from 1996 to 2002 [3]. Transgenic corn with Bt toxins active against either coleopteran pests or both coleopteran and lepidopteran pests has been registered in the United States since 2003 [3]. The most important beetle pest targeted by Bt corn is the western corn rootworm (WCR), *Diabrotica virgifera virgifera* LeConte, which together with the northern corn rootworm, *Diabrotica barberi* (Smith and Lawrence), cost farmers in the United States approximately $2 billion annually before 2003 (P. Mitchell, personal communication). Three Bt toxins active against *Diabrotica* species (Cry3Bb1, Cry34/35Ab1, and mCry3Aa) are produced either singly or in pyramids (Cry3Bb1 + Cry34/35Ab1 and mCry3Aa + Cry34/35Ab1) by Bt corn registered now in the United States [4], [5], [6], [7], [8]. Cry3Bb1 has been the most widely planted type of Bt corn active against corn rootworms, with the area planted in the United States increasing from 0.2 million ha in 2003 to 4 million ha in 2006 and 12 million ha in 2008 [9].

Evolution of resistance is the primary threat to the continued success of Cry3Bb1 corn and other Bt crops [10], [11], [12]. Field-evolved resistance is defined as a genetically based decrease in susceptibility of a population to a toxin caused by exposure of the population to the toxin in the field [3], [13]. Although Bt crops remain effective against many pest populations, field-evolved resistance associated with increased damage to Bt crops has been reported in some populations of at least five species of major target pests, including WCR [3], [14], [15], [16], [17], [18], [19], [20], [21]. Based on field-evolved WCR resistance in Iowa [19] and related reports of “unexpected damage” to Cry3Bb1 corn in the field in Iowa, Illinois, Minnesota and Nebraska, the United States Environmental Protection Agency (EPA) [22] has recommended, “the appropriate remedial action plan be implemented for Cry3Bb1 corn in areas experiencing unexpected field damage.”

The refuge strategy has been the main approach used for delaying pest resistance to Bt crops [3], [11], but the 20% refuge requirement for Cry3Bb1 corn, as implemented, apparently has not been sufficient to substantially delay WCR resistance in some areas [19], [22], [23]. Under optimal conditions, the refuge strategy is expected to work as follows [3], [11]: The initial frequency of alleles bestowing resistance is low, so that resistant pests surviving on Bt crops are rare. Rare resistant pests that survive on Bt crops will mate with abundant susceptible pests from nearby refuges of host plants without Bt toxins. Ideally, inheritance of resistance is recessive, so the hybrid progeny from such matings will die on Bt crops, substantially slowing the evolution of resistance. This approach is sometimes called the “high-dose refuge strategy” because it works best if the dose of toxin ingested by pests on Bt plants is high enough to kill all or nearly all of the hybrid progeny of resistant and susceptible insects. The success of the strategy is enhanced if resistance is associated with a fitness cost, so that on plants without Bt toxins active against the pest, individuals with alleles conferring resistance are less fit than susceptible individuals [24]. The refuge strategy also works better if fitness of resistant individuals is lower on the Bt crop than on the corresponding non-Bt crop, which is called incomplete resistance [12].

To evaluate the risk of resistance to Bt corn and the potential success of the refuge strategy, WCR has been selected for resistance to corn producing Cry3Bb1, Cry34/35Ab1 or mCryA in greenhouse and laboratory experiments [25], [26], [27], [28]. The results show that without refuges, resistance has evolved rapidly to each of the three rootworm-active toxins [25], [26], [27], [28]. The results suggest that the optimal conditions for success of the refuge strategy are not met for Cry3Bb1 corn against WCR. In particular, results of a greenhouse selection started with WCR collected in southwestern Kansas in 2002, before Cry3Bb1 corn was registered, showed that inheritance of resistance to Cry3Bb1 corn was not recessive [27]. In addition, the initial resistance allele frequency estimated from this experiment is 0.2 [29], which is 200 to 2000 times higher than the “standard” estimates of 0.001 to 0.0001 used in many resistance management models for WCR [29], [30], [31].

The success of any plans to delay or counter field-evolved resistance will depend on better understanding of WCR resistance to Cry3Bb1 corn. Toward that end, we selected WCR for resistance by rearing them in the greenhouse on roots of Cry3Bb1 corn and allowing the survivors to mate and produce offspring to start the next generation. We selected three colonies derived from the field in 2005: one each from Kansas (KS), Minnesota (MN), and Wisconsin (WI) (see Methods). We tested each of the three selected colonies and their unselected parent (control) colonies on Cry3Bb1 corn (referred to hereafter as “Bt corn”) and on similar corn that did not produce Cry3Bb1 (referred to hereafter as “isoline corn”) (see methods). Both greenhouse and field tests indicate rapid evolution of resistance to Bt corn in the selected strains.

## Results

### Greenhouse Tests of Larval and Adult Performance on Bt Corn and Isoline Corn

**Figure 1:**
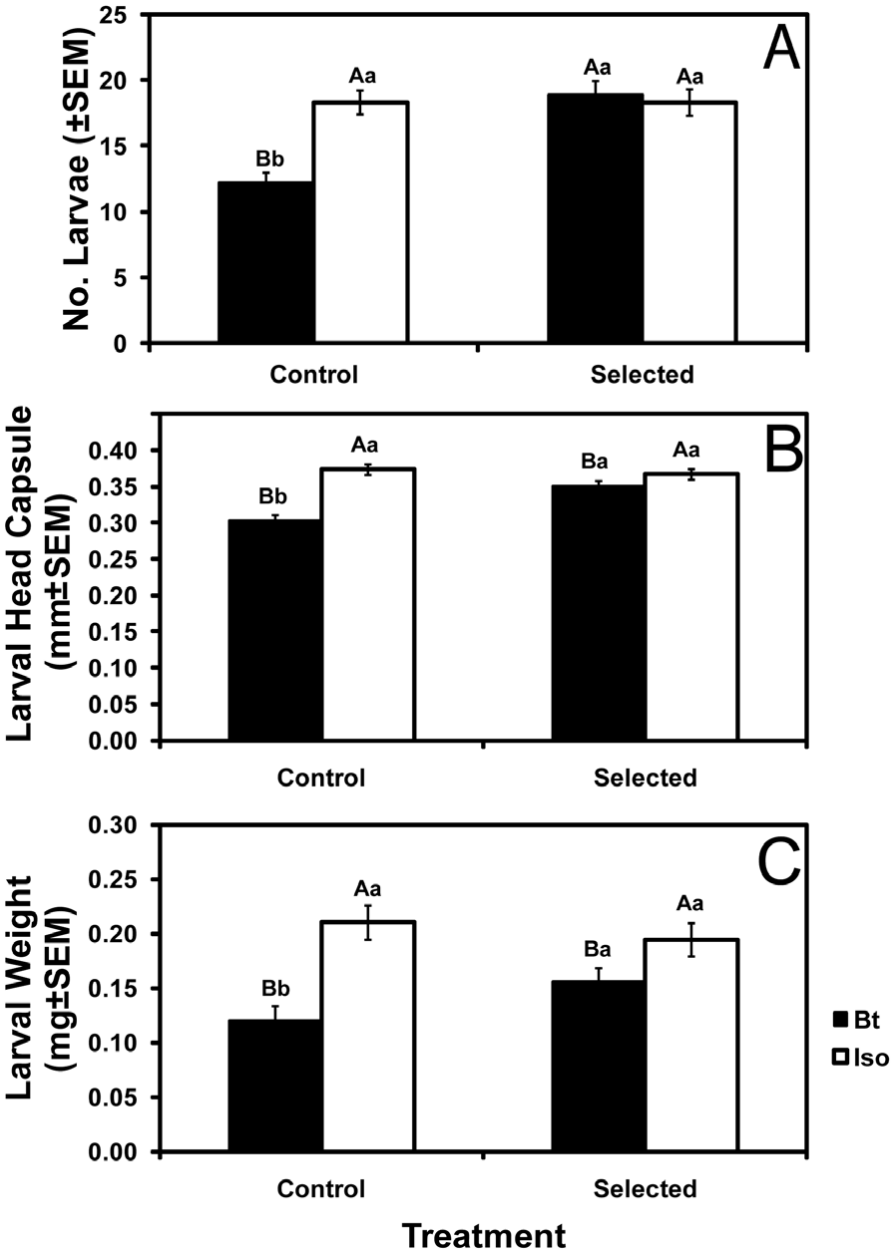
Larval performance in the greenhouse. Mean number (A), head capsule width (B), and dry weight (C) of larvae recovered 1 and 2 weeks after egg hatch in greenhouse tests conducted after three generations of greenhouse selection. Bars with the same letters are not significantly different (*P*>0.05). Capital letters indicate comparisons between isoline and Bt corn within control or selected colonies. Lowercase letters indicate comparisons between colonies within isoline or Bt corn.

In greenhouse tests conducted after three generations of selection, larval performance on Bt corn 1 and 2 weeks after egg hatch was better for selected colonies than for control colonies in terms of survival, head capsule width, and weight (Fig. 1 and S1). Larval performance on Bt corn relative to isoline corn was better for the selected colonies than for the control colonies, as reflected in a significant interaction between corn type (Bt versus isoline) and treatment (selected versus control colonies) for larval survival, head capsule width, and weight (Figs. 1 and S1; Table S1, ANOVA, P<0.002 for each trait). Survival on Bt corn relative to survival on isoline corn was greater for selected colonies (100%) than control colonies (67%) (Table S1, *F* = 27.8; df = 1,70; *P*<0.0001). For control colonies, larval survival on Bt corn was significantly lower than on isoline corn, but for selected colonies, larval survival did not differ significantly between Bt corn and isoline corn (Fig. 1A). For both selected and control colonies, larval head capsule width and weight were lower on Bt corn than on isoline corn (Fig. 1B,C).

**Figure 2:**
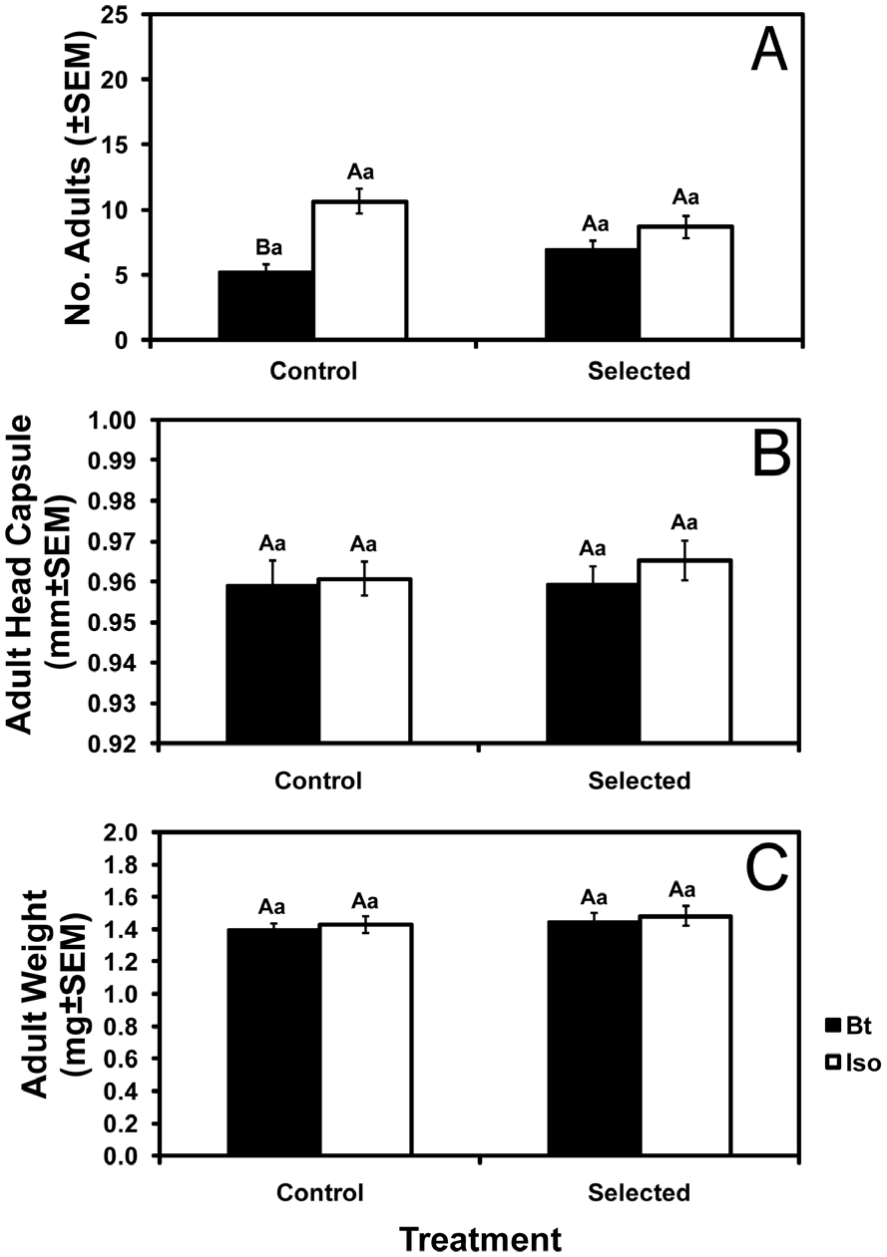
Adult performance in the greenhouse. Mean number (A), head capsule width (B), and dry weight (C) of adults from greenhouse tests conducted after three generations of greenhouse selection. Bars with the same letters are not significantly different (*P*>0.05). Capital letters indicate comparisons between isoline and Bt corn within control or selected colonies. Lowercase letters indicate comparisons between colonies within isoline or Bt corn.

As with larval survival 1 and 2 weeks after egg hatch, adult emergence on Bt corn relative to isoline corn was higher for selected than control colonies, as indicated by a significant corn type by treatment interaction (Figs. 2A and S2A, Table S2, *F* = 7.86; df = 1,154; *P* = 0.0057). However, adult emergence, adult head capsule width, and adult weight did not differ significantly between selected and control colonies on Bt corn (Fig. 2B,C). Also, adult head capsule width and weight did not differ significantly between Bt corn and isoline corn for either control or selected colonies (Figs. 2B, C) and the corn type by treatment interaction was not significant for these two adult traits (Table S2). Based on data obtained from routine rearing, the percentage of eggs that hatched did not differ significantly between control colonies reared on isoline corn and selected colonies reared on Bt corn (Table S2).

### Field Tests of Larval Performance and Root Damage for Bt Corn and Isoline Corn

**Figure 3:**
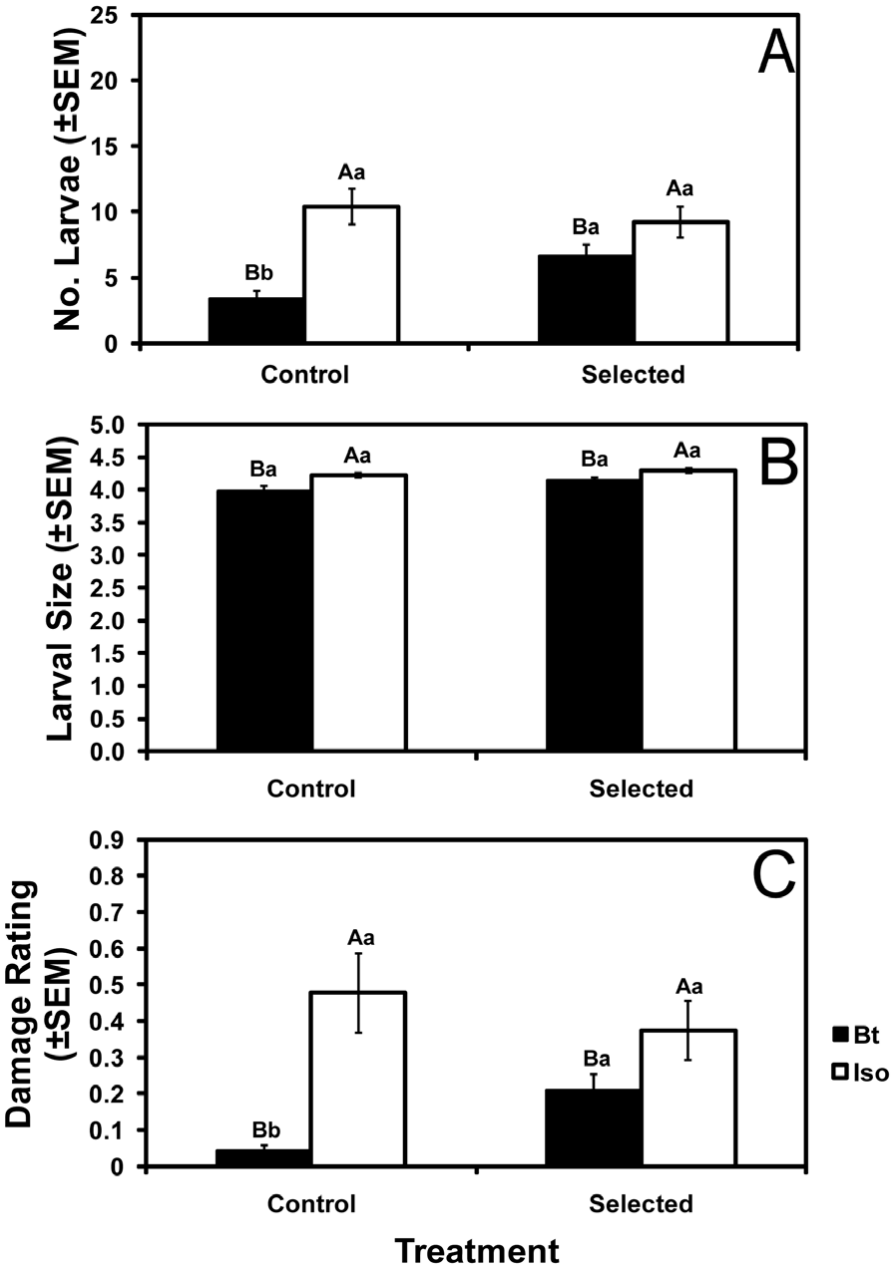
Larval performance and root damage in the field. Mean number (A) and size category (see methods) (B) of larvae recovered, and root damage ratings (C) in field tests conducted after six generations of greenhouse selection. Bars with the same letters are not significantly different (*P*>0.05). Capital letters indicate comparisons between isoline and Bt corn within control or selected colonies. Lowercase letters indicate comparisons between colonies within isoline or Bt corn.

In field tests after four to seven generations of greenhouse selection, larval survival on Bt corn was significantly higher for selected colonies than control colonies (Fig. 3A). Similar to results from the greenhouse experiments, larval survival on Bt corn relative to isoline corn was higher for the selected colonies (72%) than for the control colonies (33%) (*F* = 9.34; df = 1,78; *P* = 0.0031) (Table S3), as also reflected in a significant corn type by treatment interaction for larval number (*F* = 5.34; df = 1,17; *P* = 0.034, Table S3). Root damage was significantly higher for selected than control colonies on Bt corn, but not on non-Bt corn (Fig. 3C), yielding a significant corn type by treatment interaction (*F* = 11.5; df = 1,82; *P* = 0.0011, Table S3). Larval size did not differ significantly between control and selected colonies on Bt corn (Figs. 3B and S3). Larvae from both selected and control colonies had significantly lower survival and size on Bt corn than on isoline corn (Figs. 3A–B and S3).

### Tests for Fitness Costs on Isoline Corn

**Figure 4:**
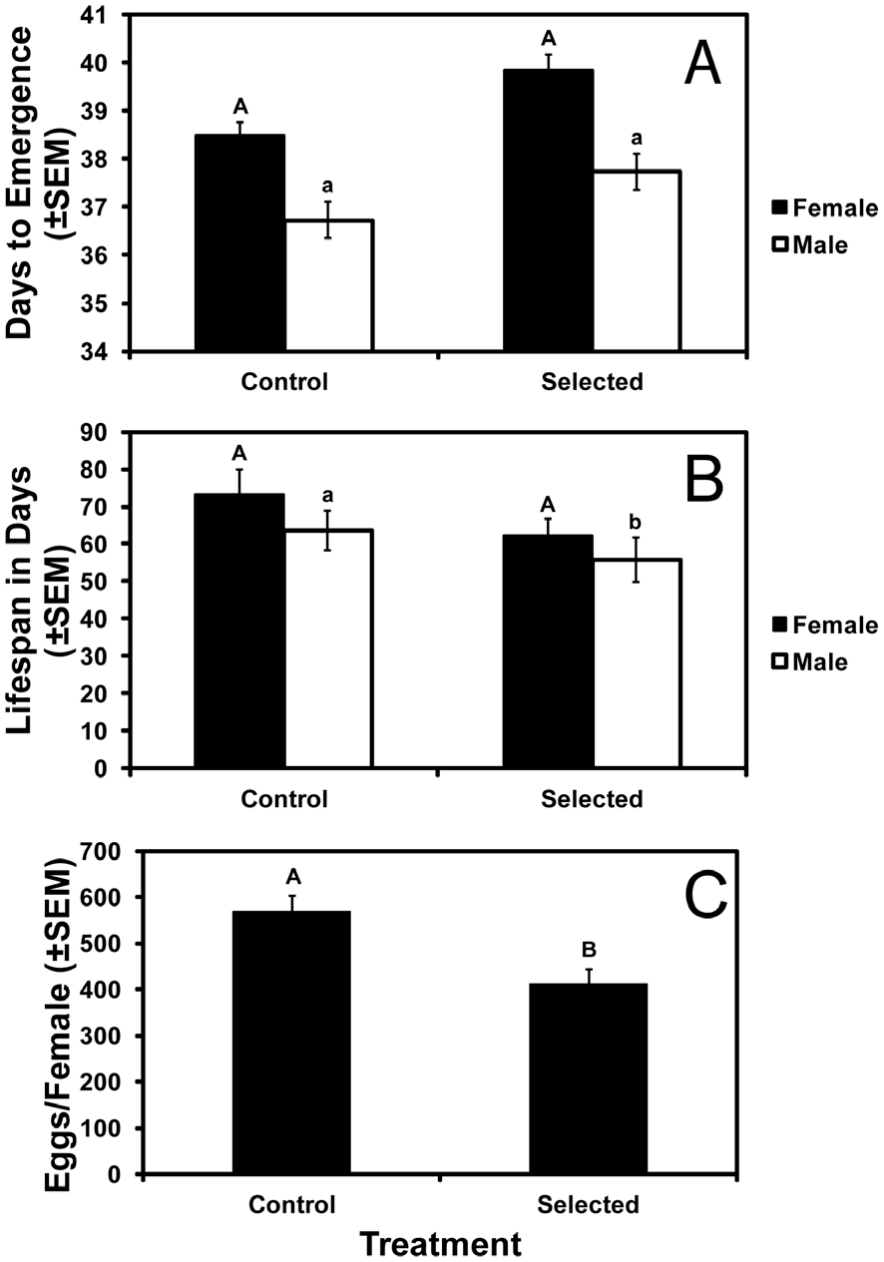
Adult performance on isoline corn. Days to emergence (A), lifespan (B), and fecundity (C) of adults. Bars with the same letters are not significantly different (*P* = 0.05). Capital letters indicate comparisons between control and selected colonies for females and lowercase letters indicate comparisons between control and selected colonies for males.

To test for fitness costs, we compared the performance of control and selected colonies in three sets of experiments, the greenhouse and field experiments described above and a third set of experiments that included greenhouse and laboratory tests (Methods). In the greenhouse and field experiments summarized above, we detected no fitness costs affecting survival, head capsule width, or weight of larvae or adults (Figs. 1–3). In the third set of experiments, we also detected no fitness costs affecting larval survival to the adult stage (6.9±1.0 adults emerging for control vs. 6.3±1.1 adults emerging for selected), adult head capsule width (20.2±0.19 mm for control vs. 20.4±0.16 mm for selected), adult weight (1.72±0.12 mg for control vs. 1.92±0.11 mg for selected), adult emergence time, adult female lifespan, or the percentage of eggs that hatched (Figs. S4, S5, Table S4). However, in the third set of experiments, we detected fitness costs on isoline corn that significantly reduced the number of eggs laid and adult male lifespan of selected colonies relative to control colonies (Fig. 4, Table S4).

To estimate the overall fitness difference between selected and control colonies on isoline corn, we calculated the potential rate of increase per generation based on eggs laid per female, the hatch rate of eggs, survival to adulthood, and percentage of adults that were female (Table 1). The potential rate of increase per generation on isoline corn was 66% for selected colonies relative to control colonies, with most of this overall fitness cost (72%) associated with egg production/female for selected colonies relative to control colonies (Table 1). The overall estimate of fitness cost does not include the potential effects of shorter male lifespan for selected colonies (56 days) relative to control colonies (64 days) (Fig. 4B).

**Table 1:** Fitness components and potential rate of increase per generation for control and selected colonies on isoline corn.

	Colony Type		
Trait	Control	Selected	Selected/Control
Fecundity (eggs per female)	570±34	410±28	0.72
Hatch Rate (%)	85±2	85±1	1.00
Survival to Adulthood (%)	28±4	25±5	0.80
Females (%)	62±5	64±6	1.03
**Growth per generation**	**84.10**	**55.80**	**0.66**

Values are means ± SEM. See Table S5 for colony generation information.

## Discussion

We found that greenhouse selection increased survival on transgenic corn producing Cry3Bb1 for three WCR colonies started in 2005 from Kansas, Minnesota, and Wisconsin, respectively. Despite initial collapses in two of the selected colonies, which may have been caused by relatively low population sizes, each of the three selected colonies evolved significant resistance to Bt corn within three generations of greenhouse selection. These results are similar to previous results showing that resistance to Cry3Bb1 corn evolved rapidly in a greenhouse-selected WCR colony derived in 2002 from a different collection site in Kansas (150 km from the collection site used here) [27] and in five replicate lab-selected WCR colonies, each derived from the same composite strain that was started by pooling individuals collected in 2004 from four states (Ohio, Colorado, Illinois and South Dakota) [28]. In the field in Iowa, WCR resistance to Cry3Bb1 corn was first documented in 2009 [19] and confirmed with additional evidence in 2010 and 2011 [32], [33]. Field derived resistance to Cry3Bb1 corn has also been documented in two counties in Illinois [34]. In addition, resistance is considered the most likely cause of unexpected damage to Cry3Bb1 corn in the field in Minnesota, Nebraska and South Dakota [35]. Collectively, the data from the laboratory, greenhouse and field demonstrate that the ability to evolve resistance to Cry3Bb1 corn is common in WCR populations.

We found that selected colonies performed better on isoline corn than on Cry3Bb1 corn. In the field, mean survival on Cry3Bb1 corn relative to isoline corn was 72% for the three selected colonies studied here (Fig. 3A) and 44% in the “constant” selected colony studied previously [27]. In previously reported laboratory bioassays with WCR from populations with field-evolved resistance, mean survival on Cry3Bb1 corn relative to non-Bt corn was 52% for 2009 populations [19] and 74% for 2010 populations [36]. The disadvantage of the selected colonies on Cry3Bb1 corn relative to isoline corn seen here and in some previous studies could reflect incomplete resistance of resistant individuals, a mixture of susceptible and resistant individuals in the selected colonies, or both [19]. Field data from Iowa in 2011, however, do not reflect incomplete resistance because the mean number of adult corn rootworm beetles emerging was not lower for Cry3Bb1 corn than for non-Bt corn [32].

Consistent with most previous studies [19], [27], [28], [36], we found no fitness costs on isoline corn that reduced survival or size of larvae or adults from selected colonies relative to control colonies. In contrast with previous results, however, we found significantly lower fecundity of selected females compared with control females on isoline corn (Fig. 4C). The trend of lower fecundity for selected versus control females occurred in all three pairs of colonies (Fig. S5) and was highly significant overall (P<0.01, Table S4). Yet, the pairwise comparison for each state (origin) considered separately was significant only for Kansas (Fig. S5). We also found a potential fitness cost in the shorter lifespan of adult males from selected colonies relative to control colonies, though the observed difference (8 d) may not be biologically relevant since most mating occurs early in the males' lifetimes [37].

In contrast to our data, Oswald et al. [36] found that fecundity was not significantly lower for WCR strains selected for resistance to Cry3Bb1 relative to control strains. In addition, unlike our results indicating that overall fitness on isoline corn was 66% for selected colonies relative to control colonies (Table 1), Oswald et al. [36] found that their estimate of overall fitness on isoline corn was not lower for selected strains compared with control strains. Also supporting the conclusion of no overall fitness cost associated with WCR resistance to Cry3Bb1, Meihls et al. [27] reported that six generations of rearing a selected colony on isoline corn did not cause major declines in resistance. Whereas no fitness costs were detected in most previous studies [19], [27], [28], [36], [38], survival on non-Bt corn was lower for 2010 populations with field-evolved resistance than for control populations in one of two tests [33]. Because most of the data indicate no fitness costs are associated with resistance to Cry3Bb1, it is prudent for the time being to infer that major fitness costs are not present in field populations. Thus, fitness costs probably will not help to substantially delay WCR resistance or to restore susceptibility if exposure to Cry3Bb1 is stopped as part of a remedial action plan.

The resistance management guidelines as implemented for WCR have been a relative failure, with field-evolved resistance to Cry3Bb1 corn documented in some Iowa populations in 2009 [19], which was the seventh year this product was registered and only the third year this type of corn was planted on >5 million hectares in the United States [9], [23]. Research has shown that several conditions favoring success of the high dose-refuge strategy do not apply for WCR and Cry3Bb1 corn. For WCR, the concentration of Cry3Bb1 in corn roots does not meet the high-dose criterion [8], [39] and resistance is not inherited recessively [27], [28], [38]. The initial resistance allele frequency was also much higher than had been assumed [29]. In light of these suboptimal conditions and the rapid evolution of resistance to Cry3Bb1 corn in many colonies selected in greenhouse and laboratory experiments [27], [28] [Fig. S1], it is not surprising that resistance evolved rapidly in the field [19], [32], [33].

Whereas this study and most previous work on WCR resistance to Bt corn has focused on Cry3Bb1 corn, WCR has also evolved resistance rapidly in selection experiments with Bt corn producing either Cry34/35Ab1 or mCry3A [25], [26]. Thus, the limited available evidence suggests that the risk of WCR resistance may be similar for Bt corn producing Cry3Bb1, Cry34/35Ab1, or mCry3A. Results of selection experiments directly addressing the risk of resistance to Bt corn producing “pyramids” of two toxins targeting WCR, which include Cry3Bb1 + Cry34/35Ab1 and mCry3A + Cry34/35Ab1 are not yet available. Pyramided Bt corn is most effective when the two toxins act independently and no cross-resistance occurs between them [40], [41]. Although resistance is generally expected to evolve slower to such two-toxin plants than to one-toxin plants, resistance to Cry3Bb1 would reduce the expected advantage of Bt corn producing Cry3Bb1 + Cry34/35Ab1 [29], [42]. For Bt corn producing Cry3Bb1 + Cry34/35Ab1, the two toxins do not appear to act independently [23].

The first resistance management plan for rootworm-active Bt corn required planting a 20% block or strip of non-Bt corn within or adjacent to the 80% Bt corn [8]. This same basic plan was used for the first three registrations (Cry3Bb1, Cry34/35Ab1, and mCry3A) [43]. In 2009, a pyramid of Cry3Bb1 and Cry34/35Ab1 was registered with just a 5% block or strip rootworm refuge required within or adjacent to the Bt corn [44]. In addition, a seed mix refuge was registered for the first time in 2010, with a 10% blend of non-Bt seed in the seed bag to serve as the refuge seed for Cry34/35Ab1 corn [5]. In April 2011, the pyramid of Cry3Bb1 + Cry34/35Ab1 was registered with a 5% seed mix refuge [4]. Finally, a pyramid of mCry3A and Cry34/35Ab1 was also registered in 2011 with a 5% block/strip [6].

The reductions in refuge percentage for seed mixes have been questioned, because smaller refuges can accelerate resistance evolution [23], [29], [45]. Moreover, the number of beetles produced by refuge plants is lower for seed mixes than for block refuges [46]. Also, research suggests that resistance may evolve quickly when larvae move from refuge corn to Bt corn when the roots of refuge plant roots have been eaten [27]. In light of rapid evolution of resistance to Cry3Bb1 corn in the laboratory, greenhouse and field, increases in the refuge percentage should be considered to substantially delay resistance in areas where field-evolved resistance has not yet occurred [23]. The US EPA has also recommended implementation of remedial actions to combat resistance to Cry3B1 corn in areas where resistance has been detected in the field [22]. Although we are not aware of any published reports of field-evolved resistance to Bt corn producing toxins other than Cry3Bb1, we recommend, as suggested by Porter et al. [35], planting non‐Bt corn, avoiding the prophylactic planting of Bt corn, and rotating Bt corn hybrids to expose rootworms to different Bt toxins. Sustainable use of Bt hybrid technology requires an integrated approach that is not overly reliant on any single tactic.

## Materials and Methods

### Origins of Insect Colonies

During the summer of 2005, French Agricultural Research (Lamberton, MN) collected eggs from feral WCR populations near Seneca, KS (in northeastern KS, ca. 150 km from the collection site in Dodge City, KS of Meihls et al. [27]), Caledonia, MN, and Janesville, WI. We refer to these sites by state (KS, MN, and WI) as the origin for each colony. We purchased 10,000 eggs from each origin, reared the larvae on isoline corn, and conducted reciprocal crosses with adults from a non-diapausing colony [47] to increase the number of generations per year from one to at least four. The wild-type genes were introgressed because the non-diapausing colony has been maintained in the laboratory for more than 200 generations and has lost genetic variation [48]. Combining eggs from the two reciprocal crosses yielded 1,357 adults from MN, 2,448 adults from WI and 8,220 adults from KS. These adults laid a total of 140,000, 264,000, and 358,000 eggs, respectively, from which larvae were split into two colonies from each origin: control colonies reared on isoline corn and selected colonies reared on Bt corn.

### Rearing and Selection

Rearing in the greenhouse and laboratory was done as described previously [26], [27]. Greenhouse air temperature was recorded hourly in each experiment (HOBO, model H08-001-02, Bourne, MA). During the screening of progeny after three generations of selection, hourly air temperatures in the greenhouse averaged 27.2±0.13°C SEM (range 13.3°C to 46.9°C), 23.2±0.07°C (range 16.4°C to 35.3°C), and 27.5±0.07°C (range 19.4°C to 52.4°C) for the MN, WI, and KS colonies, respectively. Soil temperatures likely did not vary as extensively as the air temperature, ensuring an adequate temperature for development [49], [50]. Temperature peaks normally occurred in the mid afternoon, when greenhouse cooling systems would become overwhelmed. As adults were collected and moved to the laboratory daily, they were not subjected to such extreme temperatures for long periods of time.

We reared larvae in the greenhouse using two methods, depending on available space during a particular generation. One method involved large beds (1.2 m wide ×7.5 m long ×25 cm deep) of the growth medium in which isoline or Bt corn was planted. Each Bt plant was initially infested with ∼200 eggs at ∼V2 stage [51] during the first few generations with egg hatch at ∼V4–5. In later generations this number was reduced to 100 eggs per plant (two, one, and four generations of selection for the MN, WI, and KS, respectively). Beds were covered with fine mesh screen to prevent adult escape 5–6 weeks following infestation, depending on temperature. In the second method, 500 eggs were placed in 15 cm×10 cm oval containers (708 ml, The Glad Products Company, Oakland, CA) along with ∼45 corn seeds (Bt corn for the selected colonies and isoline corn for the control colonies). Containers were then filled ∼4 cm deep with a growth medium of 2∶1 autoclaved soil and ProMix™ (Premier Horticulture Inc., Quakertown, PA). After 21 d, we cut the living corn at the soil surface and transferred the remaining contents upside down to a 33 cm×19 cm container (5.7 L, Sterilite Corporation, Clinton, SC) with new growth medium and ∼115 seeds (4 d after germination) to allow larvae to complete development and pupate.

Adults from all colonies were collected from rearing containers six times weekly. We held adults in the laboratory under 14∶10 [L∶D] photoperiod and 25°C in cages (30×30×30 cm, MegaView, Taichung, Taiwan) and provided them with artificial diet [52], fresh isoline corn leaves, and water. The oviposition substrate, consisting of 1 cm deep moist soil that had been sifted through 70 mesh (212 µm) sieves, was provided in Petri dishes. We then scarified the soil surface to promote oviposition [53]. Dishes were replaced weekly and eggs recovered by rinsing the soil through a 60 mesh sieve (250 µm) with water.

Whereas selection for resistance by rearing on Bt corn plants in the greenhouse proceeded smoothly for the KS selected colony, the MN and WI selected colonies collapsed after three and two generations of rearing on Bt corn, respectively. For MN and WI, we began selection again using the control colony for each of these origins, after six and four generations of rearing on isoline corn for MN and WI, respectively (Table S5). To provide enough individuals to maintain the selected colonies and to conduct evaluation experiments, we reared each of the three selected colonies on isoline corn in some generations (Table S5).

### Greenhouse Tests of Larval and Adult Performance on Bt Corn and Isoline Corn

After each of the selected colonies had been reared on Bt corn for three generations, we tested the performance of each pair of colonies (selected and control from each origin) simultaneously on Bt corn and isoline corn in the greenhouse (Table S5). For each replication of each treatment, three pots were planted with two corn seeds each. Two of the pots were smaller clay pots (3.3 L) for larval recovery and one pot was a larger plastic pot (9.9 L) for adult recovery. Following germination, seedlings were thinned to one plant per pot. The same growth medium was used as for rearing. To prevent larval escape, we fitted drainage holes on all pots with 114-µm stainless steel mesh (TWP Inc., Berkley, CA). Plants were watered as needed and fertilized ∼6 wk after planting with 1.25 ml of Peters Professional® Multi Purpose 20-20-20 (The Scotts Company LLC, Marysville, OH).

Three weeks after planting, we infested pots with 50 WCR eggs suspended in 0.15% agar (CAS# 9002-18-0, USB Corporation, Cleveland, OH) solution via a pipetter into a 2.5 cm deep hole in the soil. After infestation, holes were covered and the plants lightly watered. To monitor time to hatch and egg viability, a subsample of eggs was placed on moist filter paper in a Petri dish and placed near the pots. Using these data as a guide, we recovered larvae from pots either 1 or 2 weeks after peak egg hatch (larval recovery dates one and two). Larval recovery was accomplished using modified Berlese funnels equipped with a 60 W incandescent light bulb. We cut the plants near their base, emptied the pot into the funnel, and gently broke up the root ball to encourage drying. Larvae were collected in attached 450 ml jars filled with 2.5 cm water. After 2 and 4 d, larvae were collected and immediately transferred to 95% ethanol where they were counted and head capsule width measured. Following desiccation in an oven (Thelco model 16, GCA/Precision Scientific Co., Chicago, IL), larval dry weight was obtained (scale model AB135-S FACT, Mettler Toledo Inc., Columbus, OH).

The above ground portions of the corn plant in adult emergence pots were passed through a hole in insect netting, which was secured around the stalk with a cable tie and to the pot with a rubber band. Pots were checked for adults three times weekly until no adults were collected for two consecutive weeks. Recovered adults were stored in 95% ethanol until they could be counted, gender determined, and dry weight taken as described for larvae. The larval recovery experiment was a randomized complete block split-plot with the main plot being treatment and the subplot being recovery date. The adult recovery experiment was a randomized complete block. There were 15 replications for each larval recovery time and adult recovery.

### Field Tests of Larval Performance and Root Damage for Bt corn and Isoline Corn

We tested larval performance and damage to plants on Bt corn and isoline corn in field experiments at the University of Missouri Bradford Research and Extension Center. To avoid production and release of resistant adults in the field, we stopped field tests when the fastest developing larvae were second or third instars. As a result, experimental duration ranged from 20 days for replications 16–19 to 32 days for replications 10–12.

Before field tests, the number of generations of rearing on Bt corn for the selected colonies was four for MN, five for WI, and seven for KS (Table S5).

The field experiment was a randomized complete block with 20 replications. Each replicate of each treatment consisted of a single plant infested with 500 eggs from one of the above colonies. To obtain enough eggs, we reared each colony on isoline corn before the field test (Table S5). For each plant, we put the whole root ball with soil in a mesh bag (47 cm×81 cm) and hung the bag in a greenhouse. The cooling system was off in the greenhouse, which allowed the temperature to increase up to 60°C, forcing larvae to leave the hot, drying soil [54], [55]. Larvae were captured in water pans below each root ball. Larvae were collected at least twice daily. The number of WCR larvae recovered was recorded and larval size was measured on a scale of 1–5, corresponding to 2, 3, 5, 7, and 10 mm in length. Following larval recovery in the greenhouse, the dried root balls were soaked in water to remove remaining soil. Root damage was then rated using a 0–3 scale [56].

### Tests for Fitness Costs on Isoline Corn

To test for fitness costs, we compared the performance of control and selected colonies in three sets of experiments, the greenhouse and field experiments described above and a third set of experiments that included greenhouse and laboratory tests described here. In the third set of experiments, we reared larvae from all colonies on isoline corn in the greenhouse. We placed 25 neonates from each of the six colonies in oval containers of isoline corn (as described above for greenhouse rearing). Larvae were recovered from the soil via modified Berlese funnels at 4, 8, 12, 16, and 20 days post infestation. Larvae were recovered using Berlese funnels until 3^rd^ instar larvae were observed; after this time larvae or pupae were collected by hand. A treatment that continued to adult emergence was also included. Oval containers which continued until adult emergence were placed in empty plastic shoeboxes (33×19 cm; 5.7 L, Sterilite Corporation, Clinton SC) which were covered with insect netting secured with a rubber band. During the development experiment, hourly greenhouse air temperatures averaged 25.4±0.14°C (range 18.3°C to 42.5°C) when the MN and WI colonies were tested and 30.2±0.24°C (range 23.2°C to 44.9°C) when the KS colonies were tested. Recovered larvae and adults were collected into scintillation vials of 95% ethanol and their gender (for adults), head capsule width, and dry weight recorded as in the standard procedures. The experimental design was a randomized complete block with five replications.

For adult studies, we collected adults emerging during the same day from each colony and confined them in male/female pairs (30 pairs per colony) to transparent cages (5.7×5.7×7.6 cm) (Gary Plastic Packing Company, Bronx, NY). Laboratory conditions were ∼24°C, 14∶10 [L∶D] using techniques similar to Oyediran et al. [57]. Insect netting confined the insects to the cages and also provided a surface for artificial diet application [52]. Adults were fed artificial diet [52] and fresh corn leaves; water was provided via water crystals (Agrosoke International, Arlington, TX). Corn leaves, artificial diet, and water crystals were checked every other day and replaced or moistened as needed. An oviposition medium consisting of 1 cm of soil sifted through 70 mesh sieves (U.S.A. Standard Sieve Series Sieve, 212 µm) covered the bottom of the cages. The soil was scarified to promote oviposition. The oviposition medium was kept moist and checked every other day along with the food and water whereas adult mortality was monitored daily. The experimental design was a randomized complete block with 30 replications.

Females were moved to clean cages with new oviposition medium weekly. Eggs from each female were collected from the soil that had been sieved through 70 mesh (212 µm) sieves by rinsing the soil with gently running water thru a 60 mesh (250 µm) sieve, then eggs were rinsed onto filter paper (11.0 cm diameter, Whatman® #1 qualitative, Florham Park, New Jersey) using a Buchner funnel. Egg production per female was recorded weekly. All eggs from a colony/treatment combination were then pooled, and a subset of 300 eggs was removed for viability testing. The 300 eggs for viability testing were placed (100 per dish) on moistened filter paper (9.0 cm diameter, Whatman® #1 qualitative, Florham Park, New Jersey) in a Petri dish (100×15 mm, Fisherbrand®, Pittsburg, Pennsylvania) and sealed with Parafilm® M (5 cm width, Pechiney Plastic Packaging, Menasha, WI). Eggs were inspected daily for hatch.

### Statistical Analysis

Although non-transformed data are shown in the figures, data were transformed as indicated by the Box-Cox method of power transformation in the SAS statistical package prior to analysis to meet the assumptions of the analysis [58], [59]. Normality of residuals was checked by observing skewness, kurtosis, and tests for normality produced by PROC UNIVARIATE of the SAS statistical package [60].

#### Greenhouse Experiments

Larval data were analyzed as a randomized complete block four way factorial arrangement (three origins × two treatments × two corn types × two larval recovery times) using PROC MIXED of the SAS statistical package [60]. The model contained the main effect of origin, treatment, corn type, larval recovery time, and all possible interactions. Replications and replication within origin, treatment, corn, and recovery time were included as the random variable. A separate analysis was done for number of larvae recovered (*x* ^0.65^), larval head capsule width (square root transformed), and larval weight (*x* ^0.25^). Adult data were analyzed separately as a randomized complete block design using PROC MIXED. The adult model contained the effect of origin, treatment, corn type, and all possible interactions. Replications and replication within origin, treatment, and corn were included as the random variable. A separate analysis was done for number of adults recovered (untransformed), adult head capsule width (square root transformed), and adult weight (log transformed). Egg viability data were analyzed as a randomized complete block design using PROC GLIMMIX (dist = beta, link = logit) of the SAS statistical package. The model contained the effect of origin, treatment, and all possible interactions.

#### Field Experiment

Larval data were analyzed as a randomized complete block three way factorial arrangement (three origins × two treatments × two corn types) using PROC MIXED. The model contained the main effect of origin, treatment, corn type, and all possible interactions. Replications and replication within origin, treatment, and corn were included as the random variable. A separate analysis was done for number of larvae recovered (*x* ^0.35^) and larval length (*x* ^2.45^).

Root damage ratings were log transformed (*x* ^0.15^) prior to analysis to meet the assumptions of the analysis. Damage ratings were analyzed as a randomized complete block three way factorial arrangement (three origins × two treatments × two corn types) using PROC MIXED. The model contained the effect of origin, treatment, corn type, and all possible interactions. Replications and replication within origin, treatment, and corn were included as the random variable.

Using larval recovery numbers, percent survival on Bt corn relative to isoline corn was determined for each colony (Figure 4-S4). For each replication, the relative survival was determined (survival on Bt corn/survival on isoline corn). These ratios were then rank transformed to meet the assumptions of the model [58] and analyzed as a randomized complete block design using PROC MIXED of the SAS statistical package [60]. Relative survival was analyzed as a randomized complete block two way factorial arrangement (three origins × two treatments) using PROC MIXED. The model contained the effect of origin, treatment, and all possible interactions. Replications and replication within origin, and treatment were included as the random variable.

#### Development Time Experiment

Number recovered (untransformed), head capsule width, and weight (*x* ^0.15^) were analyzed separately as a randomized complete block three way factorial arrangement (three origins × two treatments × six recovery dates) using PROC MIXED. The model contained the main effect of origin, treatment, recovery date, and all possible interactions. Replications and replication within origin, treatment, and date were included as the random variable.

#### Adult Longevity, Oviposition, and Egg Viability

Total oviposition per female (*x* ^0.7^) and lifespan (untransformed) were analyzed separately as a randomized complete block two way factorial arrangement (three origins × two treatments) using PROC MIXED of the SAS statistical package [60]. The model contained the main effect of origin, treatment, and all possible interactions. Replications and replication within origin, and treatment were included as the random variable. Male and female lifespan data were analyzed separately. Days to emergence data (untransformed) were analyzed in the same way as oviposition and lifespan data. Egg viability data were analyzed as a randomized complete block design using PROC GLIMMIX (dist = beta, link = logit) of the SAS statistical package. The model contained the effect of origin, treatment, and all possible interactions.

## Supporting Information

Figure S1 **Larval recovery of individual colonies on Bt and isoline corn following three generations of greenhouse selection.** Mean number (A), head capsule width (B), and dry weight (C) of larvae recovered from laboratory colonies during trials on Bt and non-transgenic isoline corn in the greenhouse after three generations of selection. Bars with the same letters are not significantly different (*P* = 0.05). Capital letters indicate comparisons between isoline and Bt within colonies and lowercase letters indicate comparisons within an origin within treatments on Bt or isoline corn.
(TIF)

Figure S2 **Adult recovery of individual colonies on Bt and isoline corn following three generations of greenhouse selection.** Mean number (A), head capsule width (B), and dry weight (C) of beetles recovered from laboratory colonies during trials on Bt and non-transgenic isoline corn in the greenhouse after three generations of selection. Bars with the same letters are not significantly different (*P* = 0.05). Capital letters indicate comparisons between isoline and Bt within colonies and lowercase letters indicate comparisons within an origin within treatments on Bt or isoline corn.
(TIF)

Figure S3 **Larval recovery of individual colonies on Bt and isoline corn under field conditions.** Mean number (A) and size (B) of larvae, and root damage ratings (C) from trials on Bt and isoline corn in the field after ∼six generations of selection. Bars with the same letters are not significantly different (*P* = 0.05). Capital letters indicate comparisons between isoline and Bt within colonies and lowercase letters indicate comparisons within an origin within treatments on Bt or isoline corn.
(TIF)

Figure S4 **Days to beetle emergence of individual control and selected colonies reared on isoline corn.** Bars with the same letters are not significantly different (*P* = 0.05). Capital letters indicate comparisons between treatments within an origin.
(TIF)

Figure S5 **Beetle longevity and female fecundity of individual control and selected colonies reared on isoline corn.** Bars with the same letters are not significantly different (*P* = 0.05). Capital letters indicate comparisons between treatments within an origin.
(TIF)

Table S1 **Analysis of variance for larval greenhouse data following three generations of selection.** See table S5 for colony generation information.
(DOCX)

Table S2 **Analysis of variance for adult greenhouse data following three generations of** selection. See table S5 for colony generation information.
(DOCX)

Table S3 **Analysis of variance for field data following greenhouse selection.** See table S5 for colony generation information.
(DOCX)

Table S4 **Analysis of variance for fitness components of control and selected colonies.** See table S5 for colony generation information.
(DOCX)

Table S5 **Summary of type of corn (Bt or isoline) used for rearing and generations tested in experiments.** ^a^ The MN and WI selected colonies collapsed after three and two generations, respectively, of initial rearing on Bt corn. We began selection again using a subset of the control colony for each of these origins, after rearing on isoline corn for six generations for MN and four generations for WI. * Greenhouse test on Bt corn and isoline corn ** Field test on Bt corn and isoline corn † Fitness cost tests on isoline corn only for larval performance in the greenhouse and adult performance in the laboratory.
(DOCX)
