# Supplementary figures and images for: Greenhouse-Selected Resistance to Cry3Bb1-Producing Corn in Three Western Corn Rootworm Populations
Source: PLoS One. 2012 Dec 20;7(12):e51055. doi: 10.1371/journal.pone.0051055 (PMC3527414; doi:10.1371/journal.pone.0051055)

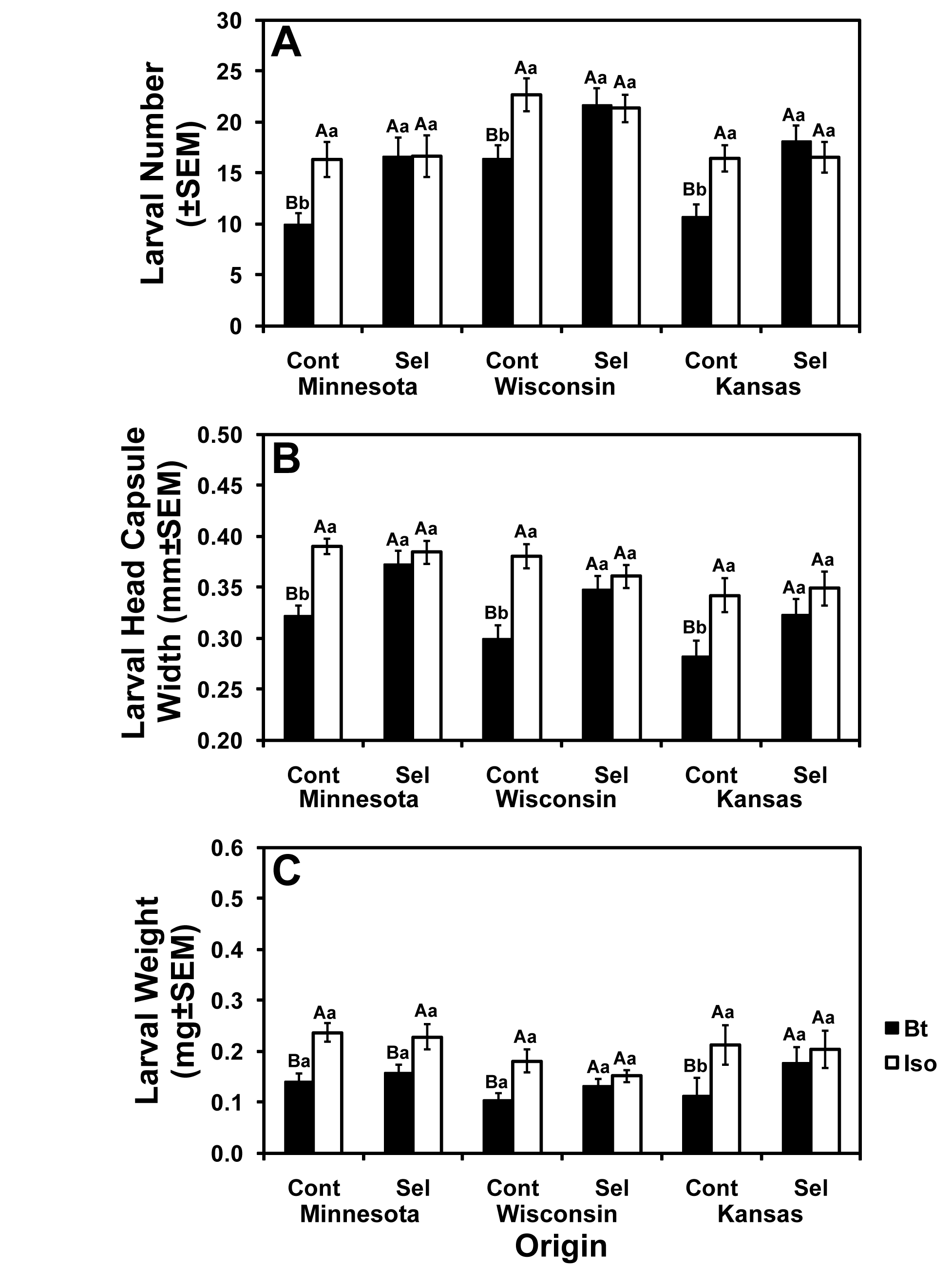

Supplement: Figure S1 — Larval recovery of individual colonies on Bt and isoline corn following three generations of greenhouse selection. Mean number (A), head capsule width (B), and dry weight (C) of larvae recovered from laboratory colonies during trials on Bt and non-transgenic isoline corn in the greenhouse after three generations of selection. Bars with the same letters are not significantly different (P = 0.05). Capital letters indicate comparisons between isoline and Bt within colonies and lowercase letters indicate comparisons within an origin within treatments on Bt or isoline corn. (TIF) [file pone.0051055.s001.tif]

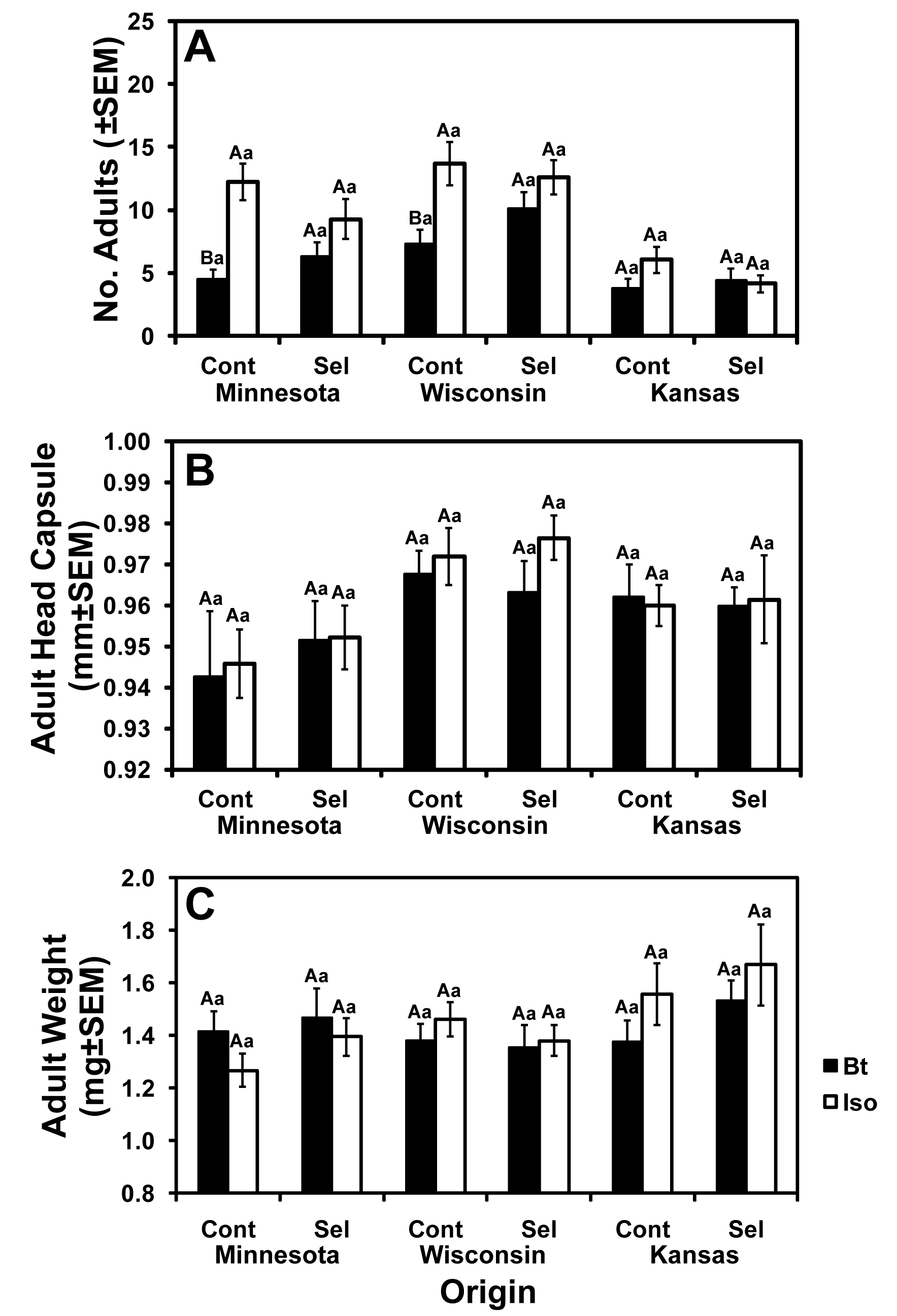

Supplement: Figure S2 — Adult recovery of individual colonies on Bt and isoline corn following three generations of greenhouse selection. Mean number (A), head capsule width (B), and dry weight (C) of beetles recovered from laboratory colonies during trials on Bt and non-transgenic isoline corn in the greenhouse after three generations of selection. Bars with the same letters are not significantly different (P = 0.05). Capital letters indicate comparisons between isoline and Bt within colonies and lowercase letters indicate comparisons within an origin within treatments on Bt or isoline corn. (TIF) [file pone.0051055.s002.tif]

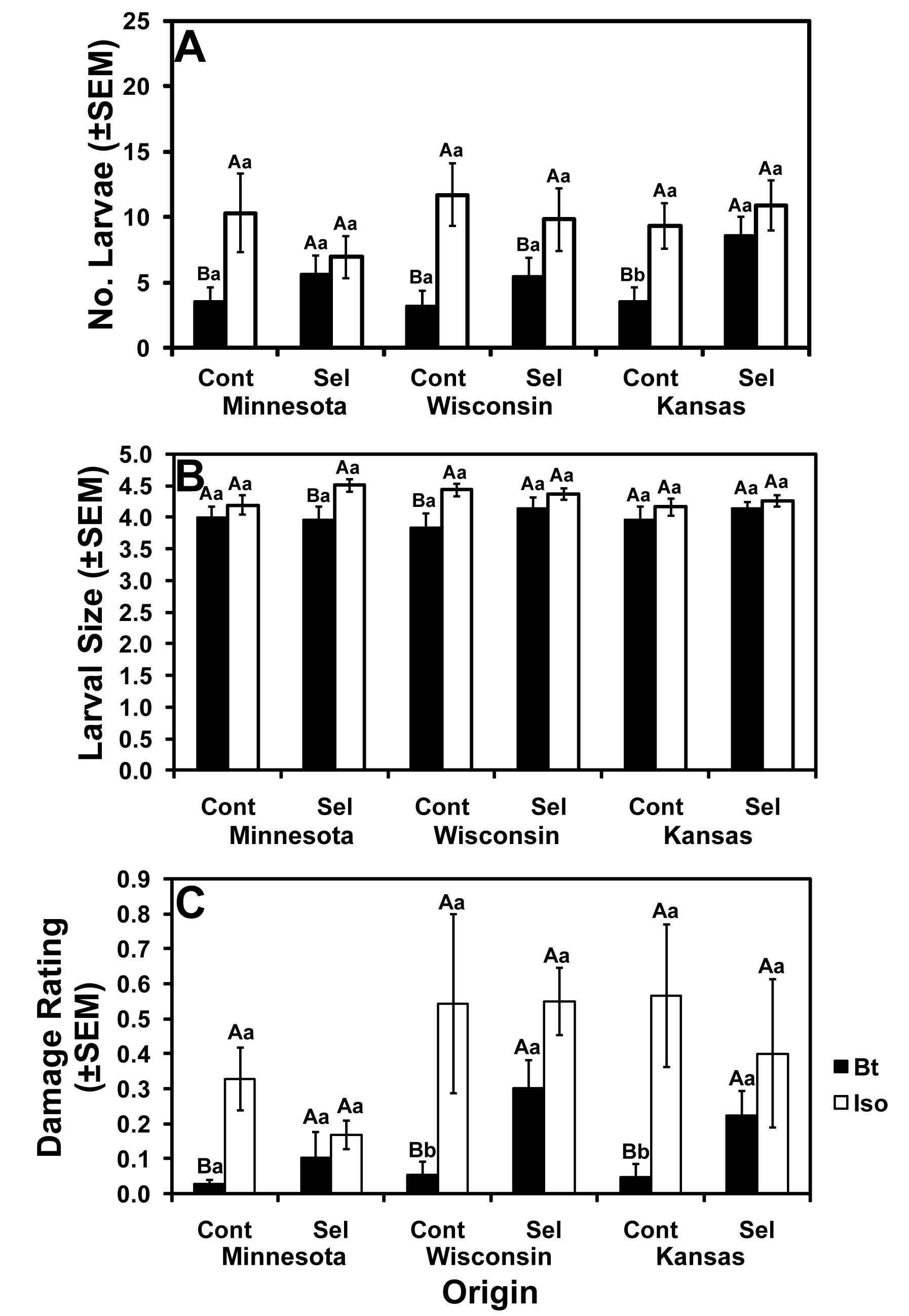

Supplement: Figure S3 — Larval recovery of individual colonies on Bt and isoline corn under field conditions. Mean number (A) and size (B) of larvae, and root damage ratings (C) from trials on Bt and isoline corn in the field after ∼six generations of selection. Bars with the same letters are not significantly different (P = 0.05). Capital letters indicate comparisons between isoline and Bt within colonies and lowercase letters indicate comparisons within an origin within treatments on Bt or isoline corn. (TIF) [file pone.0051055.s003.tif]

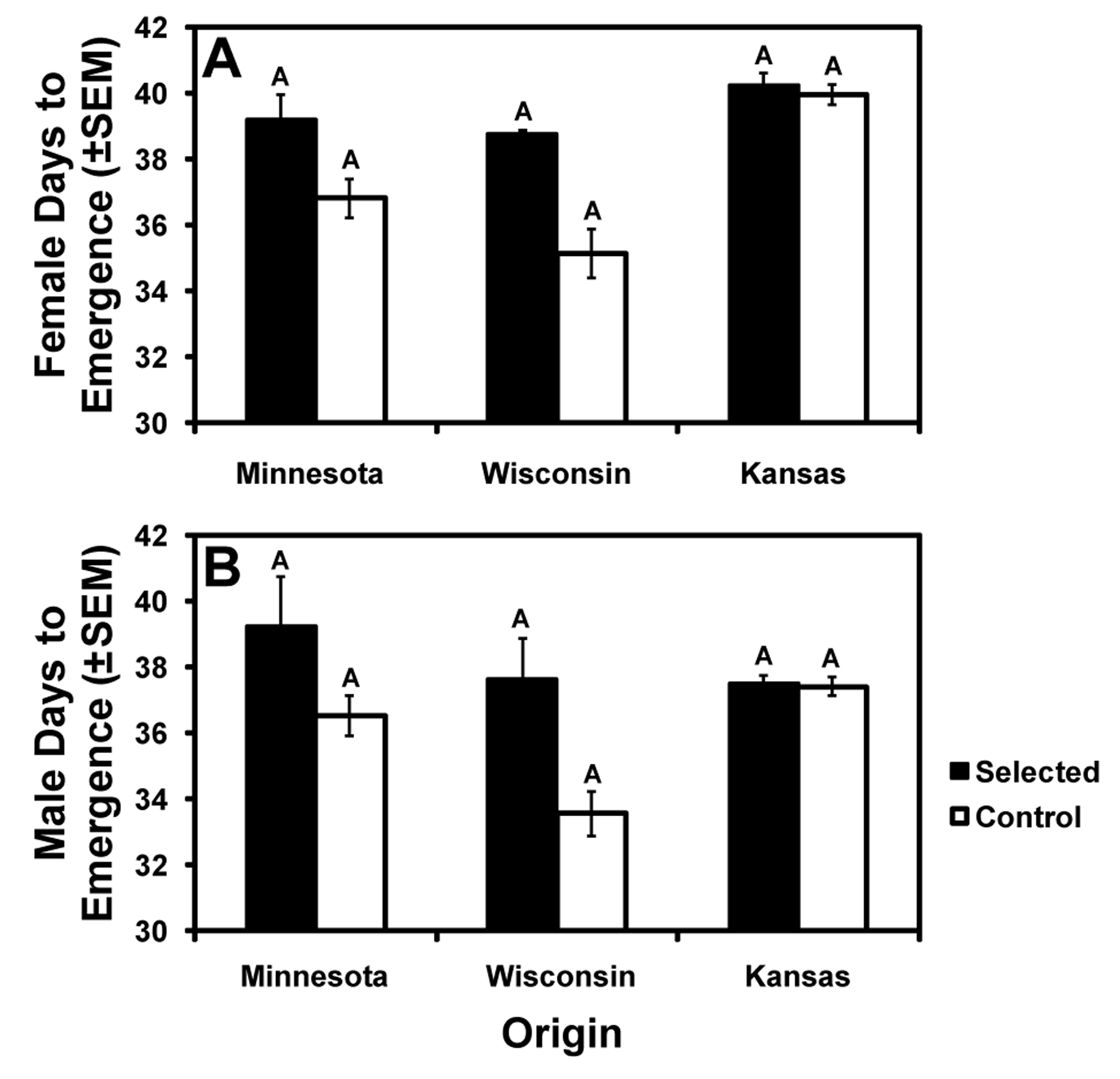

Supplement: Figure S4 — Days to beetle emergence of individual control and selected colonies reared on isoline corn. Bars with the same letters are not significantly different (P = 0.05). Capital letters indicate comparisons between treatments within an origin. (TIF) [file pone.0051055.s004.tif]

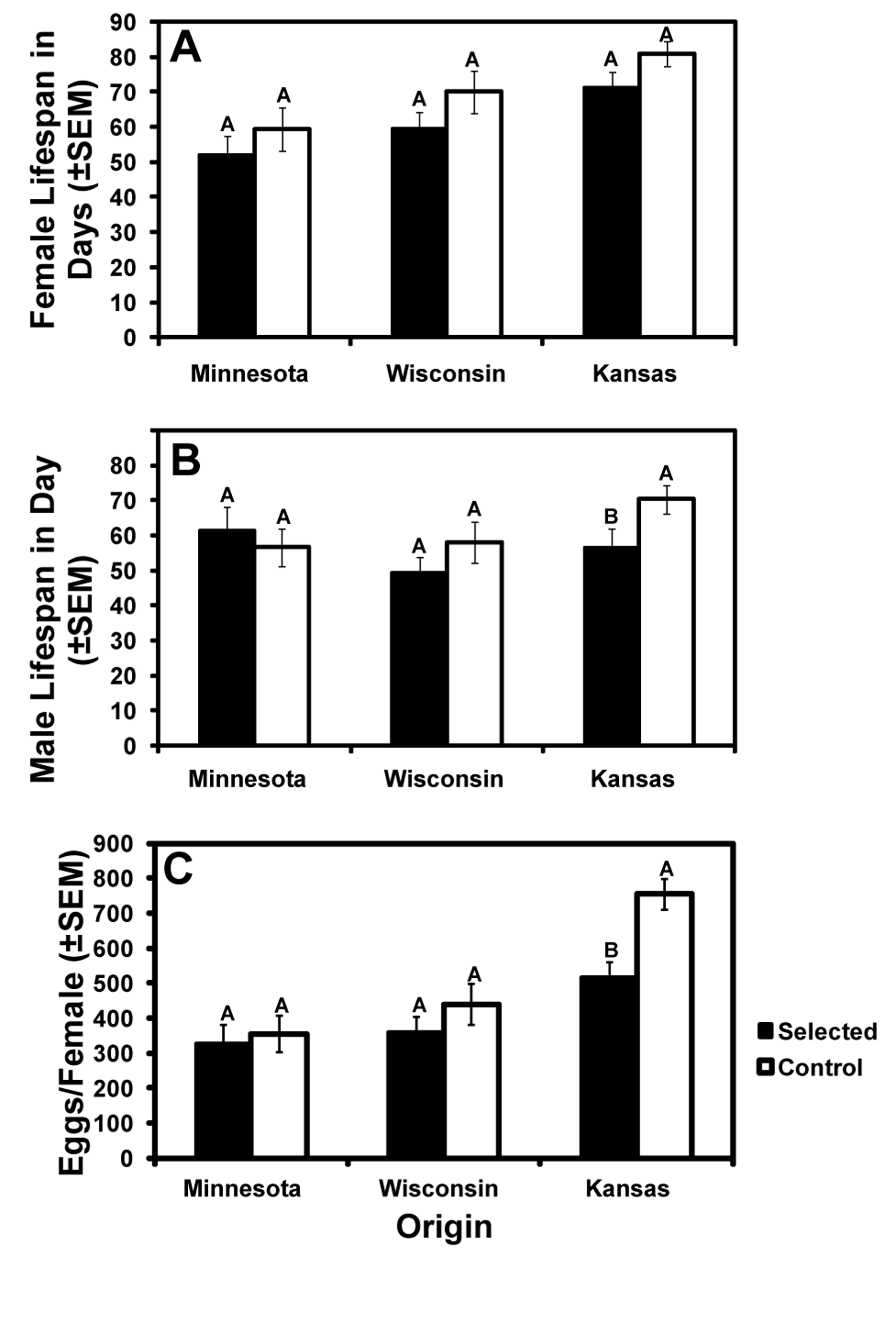

Supplement: Figure S5 — Beetle longevity and female fecundity of individual control and selected colonies reared on isoline corn. Bars with the same letters are not significantly different (P = 0.05). Capital letters indicate comparisons between treatments within an origin. (TIF) [file pone.0051055.s005.tif]
