# Supplementary material for: Greenhouse-Selected Resistance to Cry3Bb1-Producing Corn in Three Western Corn Rootworm Populations
Source: PLoS One. 2012 Dec 20;7(12):e51055. doi: 10.1371/journal.pone.0051055 (PMC3527414; doi:10.1371/journal.pone.0051055)
Supplement: Table S1 — Analysis of variance for larval greenhouse data following three generations of selection. See table S5 for colony generation information. (DOCX) [file pone.0051055.s006.docx]

**Table S1.** Analysis of variance for larval greenhouse data following three generations of selection.

| **Analysis** | **Effect** | **df** | **F value** | **P** |
| --- | --- | --- | --- | --- |
| **Larval Number** | Trt | 1,321 | 14.92 | 0.0001 |
|  | Ori | 2,321 | 20.76 | <.0001 |
|  | Ori*Trt | 2,321 | 1.36 | 0.2578 |
|  | Corn | 1,321 | 12.78 | 0.0004 |
|  | Corn*Trt | 1,321 | 16.83 | <.0001 |
|  | Ori*Corn | 2,321 | 0.14 | 0.8717 |
|  | Ori*Corn*Trt | 2,321 | 0.07 | 0.9296 |
|  | Time | 1,321 | 7.12 | 0.0080 |
|  | Trt*Time | 1,321 | 1.06 | 0.3051 |
|  | Ori*Time | 2,321 | 4.61 | 0.0106 |
|  | Ori*Trt*Time | 2,321 | 0.56 | 0.5728 |
|  | Corn*Time | 1,321 | 0.87 | 0.3524 |
|  | Corn*Trt*Time | 1,321 | 2.25 | 0.1349 |
|  | Ori*Corn*Time | 2,321 | 0.42 | 0.6555 |
|  | Ori*Corn*Trt*Time | 2,321 | 0.39 | 0.6775 |
| **Larval Head Capsule** | Trt | 1,315 | 9.99 | 0.0017 |
|  | Ori | 2,315 | 21.90 | <.0001 |
|  | Ori*Trt | 2,315 | 0.10 | 0.9011 |
|  | Corn | 1,315 | 56.47 | <.0001 |
|  | Corn*Trt | 1,315 | 21.12 | <.0001 |
|  | Ori*Corn | 2,315 | 0.16 | 0.8521 |
|  | Ori*Corn*Trt | 2,315 | 0.63 | 0.5330 |
|  | Time | 1,315 | 213.57 | <.0001 |
|  | Trt*Time | 1,315 | 0.38 | 0.5398 |
|  | Ori*Time | 2,315 | 5.45 | 0.0047 |
|  | Ori*Trt*Time | 2,315 | 0.11 | 0.8928 |
|  | Corn*Time | 1,315 | 2.61 | 0.1073 |
|  | Corn*Trt*Time | 1,315 | 0.06 | 0.8016 |
|  | Ori*Corn*Time | 2,315 | 2.55 | 0.0797 |
|  | Ori*Corn*Trt*Time | 2,315 | 1.61 | 0.2007 |
| **Larval Weight** | Trt | 1,315 | 3.41 | 0.0659 |
|  | Ori | 2,315 | 14.77 | <.0001 |
|  | Ori*Trt | 2,315 | 1.24 | 0.2903 |
|  | Corn | 1,315 | 50.45 | <.0001 |
|  | Corn*Trt | 1,315 | 9.86 | 0.0018 |
|  | Ori*Corn | 2,315 | 0.29 | 0.7488 |
|  | Ori*Corn*Trt | 2,315 | 1.50 | 0.2237 |
|  | Time | 1,315 | 169.04 | <.0001 |
|  | Trt*Time | 1,315 | 0.01 | 0.9038 |
|  | Ori*Time | 2,315 | 14.85 | <.0001 |
|  | Ori*Trt*Time | 2,315 | 0.06 | 0.9430 |
|  | Corn*Time | 1,315 | 1.93 | 0.1660 |
|  | Corn*Trt*Time | 1,315 | 0.04 | 0.8350 |
|  | Ori*Corn*Time | 2,315 | 1.31 | 0.2707 |
|  | Ori*Corn*Trt*Time | 2,315 | 1.32 | 0.2674 |
| **Relative Survival** | Trt | 1,70 | 27.81 | <.0001 |
|  | Ori | 2,70 | 0.44 | 0.6447 |
|  | Ori*Trt | 2,70 | 0.52 | 0.5951 |

See table S5 for colony generation information.
