# Supplementary material for: Greenhouse-Selected Resistance to Cry3Bb1-Producing Corn in Three Western Corn Rootworm Populations
Source: PLoS One. 2012 Dec 20;7(12):e51055. doi: 10.1371/journal.pone.0051055 (PMC3527414; doi:10.1371/journal.pone.0051055)
Supplement: Table S2 — Analysis of variance for adult greenhouse data following three generations of selection. See table S5 for colony generation information. (DOCX) [file pone.0051055.s007.docx]

**Table S2.** Analysis of variance for adult greenhouse data following three generations of selection.

| **Analysis** | **Effect** | **df** | **F value** | **P** |
| --- | --- | --- | --- | --- |
| **Adult Number** | Trt | 1,154 | 0.04 | 0.8397 |
|  | Ori | 2,154 | 30.80 | <.0001 |
|  | Ori*Trt | 2,154 | 0.54 | 0.5831 |
|  | Corn | 1,154 | 29.93 | <.0001 |
|  | Corn*Trt | 1,154 | 7.86 | 0.0057 |
|  | Ori*Corn | 2,154 | 4.07 | 0.0189 |
|  | Ori*Corn*Trt | 2,154 | 0.23 | 0.7912 |
| **Adult Head Capsule** | Trt | 1,142 | 0.23 | 0.6307 |
|  | Ori | 2,142 | 6.76 | 0.0016 |
|  | Ori*Trt | 2,142 | 0.25 | 0.7823 |
|  | Corn | 1,142 | 0.48 | 0.4881 |
|  | Corn*Trt | 1,142 | 0.18 | 0.6700 |
|  | Ori*Corn | 2,142 | 0.34 | 0.7155 |
|  | Ori*Corn*Trt | 2,142 | 0.10 | 0.9058 |
| **Adult Weight** | Trt | 1,142 | 1.03 | 0.3117 |
|  | Ori | 2,142 | 3.21 | 0.0432 |
|  | Ori*Trt | 2,142 | 1.72 | 0.1833 |
|  | Corn | 1,142 | 0.35 | 0.5548 |
|  | Corn*Trt | 1,142 | 0.00 | 0.9444 |
|  | Ori*Corn | 2,142 | 1.88 | 0.1570 |
|  | Ori*Corn*Trt | 2,142 | 0.26 | 0.7716 |
| **Percent Hatch** | Trt | 1,12 | 0.56 | 0.4684 |
|  | Ori | 2,12 | 0.21 | 0.8147 |
|  | Ori*Trt | 2,12 | 0.58 | 0.5770 |

See table S5 for colony generation information.
