# Supplementary material for: Greenhouse-Selected Resistance to Cry3Bb1-Producing Corn in Three Western Corn Rootworm Populations
Source: PLoS One. 2012 Dec 20;7(12):e51055. doi: 10.1371/journal.pone.0051055 (PMC3527414; doi:10.1371/journal.pone.0051055)
Supplement: Table S3 — Analysis of variance for field data following greenhouse selection. See table S5 for colony generation information. (DOCX) [file pone.0051055.s008.docx]

**Table S3.** Analysis of variance for field data following greenhouse selection.

| **Analysis** | **Effect** | **df** | **F value** | **P** |
| --- | --- | --- | --- | --- |
| **Larval Number** | Trt | 1,17 | 2.99 | 0.1017 |
|  | Ori | 2,17 | 1.45 | 0.2623 |
|  | Ori*Trt | 2,17 | 1.04 | 0.3764 |
|  | Corn | 1,17 | 28.24 | <.0001 |
|  | Corn*Trt | 1,17 | 5.34 | 0.0336 |
|  | Ori*Corn | 2,17 | 1.25 | 0.3113 |
|  | Ori*Corn*Trt | 2,17 | 0.23 | 0.7998 |
| **Larval Size** | Trt | 1,169 | 3.33 | 0.0697 |
|  | Ori | 2,169 | 0.94 | 0.3909 |
|  | Ori*Trt | 2,169 | 0.46 | 0.6309 |
|  | Corn | 1,169 | 17.38 | <.0001 |
|  | Corn*Trt | 1,169 | 0.44 | 0.5063 |
|  | Ori*Corn | 2,169 | 0.55 | 0.5754 |
|  | Ori*Corn*Trt | 2,169 | 1.01 | 0.3677 |
| **Root Damage** | Trt | 1,82 | 5.85 | 0.0178 |
|  | Ori | 2,82 | 4.37 | 0.0157 |
|  | Ori*Trt | 2,82 | 2.11 | 0.1274 |
|  | Corn | 1,82 | 38.70 | <.0001 |
|  | Corn*Trt | 1,82 | 11.53 | 0.0011 |
|  | Ori*Corn | 2,82 | 0.26 | 0.7718 |
|  | Ori*Corn*Trt | 2,82 | 0.47 | 0.6277 |
| **Percent Hatch** | Trt | 1,11 | 0.28 | 0.6095 |
|  | Ori | 2,11 | 7.89 | 0.0075 |
|  | Ori*Trt | 2,11 | 4.54 | 0.0364 |
| **Relative Survival** | Trt | 1,78 | 9.34 | 0.0031 |
|  | Ori | 2,78 | 1.32 | 0.2734 |
|  | Ori*Trt | 2,78 | 0.05 | 0.9486 |

See table S5 for colony generation information.
