# Supplementary material for: Greenhouse-Selected Resistance to Cry3Bb1-Producing Corn in Three Western Corn Rootworm Populations
Source: PLoS One. 2012 Dec 20;7(12):e51055. doi: 10.1371/journal.pone.0051055 (PMC3527414; doi:10.1371/journal.pone.0051055)
Supplement: Table S4 — Analysis of variance for fitness components of control and selected colonies. See table S5 for colony generation information. (DOCX) [file pone.0051055.s009.docx]

**Table S4.** Analysis of variance for fitness components of control and selected colonies.

| **Analysis** | **Effect** | **df** | **F value** | **P** |
| --- | --- | --- | --- | --- |
| **Number Recovered** | Trt | 1,111 | 2.64 | 0.1068 |
|  | Ori | 2,111 | 12.77 | <.0001 |
|  | Ori*Trt | 2,111 | 0.96 | 0.3843 |
|  | Date | 5,111 | 24.52 | <.0001 |
|  | Trt*Date | 5,111 | 1.89 | 0.1023 |
|  | Ori*Date | 10,111 | 7.76 | <.0001 |
|  | Ori*Trt*Date | 10,111 | 1.61 | 0.1140 |
| **Head Capsule Width** | Trt | 1,114 | 0.42 | 0.5171 |
|  | Ori | 2,114 | 3.07 | 0.0502 |
|  | Ori*Trt | 2,114 | 0.08 | 0.9254 |
|  | Date | 5,114 | 1339.15 | <.0001 |
|  | Trt*Date | 5,114 | 1.33 | 0.2571 |
|  | Ori*Date | 10,114 | 34.40 | <.0001 |
|  | Ori*Trt*Date | 10,114 | 0.75 | 0.6767 |
| **Weight** | Trt | 1,111 | 0.02 | 0.8855 |
|  | Ori | 2,111 | 36.64 | <.0001 |
|  | Ori*Trt | 2,111 | 0.31 | 0.7371 |
|  | Date | 5,111 | 299.01 | <.0001 |
|  | Trt*Date | 5,111 | 1.17 | 0.3277 |
|  | Ori*Date | 10,111 | 18.98 | <.0001 |
|  | Ori*Trt*Date | 10,111 | 0.39 | 0.9473 |
| **Female Days to Emergence** | Trt | 1,15 | 1.80 | 0.1993 |
|  | Ori | 2,15 | 2.98 | 0.0813 |
|  | Ori*Trt | 2,15 | 0.20 | 0.8247 |
| **Male Days to Emergence** | Trt | 1,14 | 1.91 | 0.1891 |
|  | Ori | 2,14 | 1.53 | 0.2512 |
|  | Ori*Trt | 2,14 | 0.46 | 0.6376 |
| **Female Longevity** | Trt | 1,131 | 5.00 | 0.0270 |
|  | Ori | 2,131 | 8.98 | 0.0002 |
|  | Ori*Trt | 2,131 | 0.05 | 0.9491 |
| **Male Longevity** | Trt | 1,132 | 1.77 | 0.1852 |
|  | Ori | 2,132 | 1.85 | 0.1617 |
|  | Ori*Trt | 2,132 | 1.51 | 0.2255 |
| **Eggs Per Female** | Trt | 1,148 | 6.82 | 0.0099 |
|  | Ori | 2,148 | 21.83 | <.0001 |
|  | Ori*Trt | 2,148 | 2.14 | 0.1216 |
| **Percent Hatch** | Trt | 1,128 | 2.17 | 0.1432 |
|  | Ori | 2,128 | 357.86 | <.0001 |
|  | Ori*Trt | 2,128 | 0.79 | 0.4580 |

See table S5 for colony generation information.
