# Supplementary material for: Greenhouse-Selected Resistance to Cry3Bb1-Producing Corn in Three Western Corn Rootworm Populations
Source: PLoS One. 2012 Dec 20;7(12):e51055. doi: 10.1371/journal.pone.0051055 (PMC3527414; doi:10.1371/journal.pone.0051055)
Supplement: Table S5 — Summary of type of corn (Bt or isoline) used for rearing and generations tested in experiments. a The MN and WI selected colonies collapsed after three and two generations, respectively, of initial rearing on Bt corn. We began selection again using a subset of the control colony for each of these origins, after rearing on isoline corn for six generations for MN and four generations for WI. * Greenhouse test on Bt corn and isoline corn ** Field test on Bt corn and isoline corn † Fitness cost tests on isoline corn only for larval performance in the greenhouse and adult performance in the laboratory. (DOCX) [file pone.0051055.s010.docx]

**Table S5.** Summary of type of corn (Bt or isoline) used for rearing and generations tested in experiments.

|  | **Minnesota (MN)** | | **Wisconsin (WI)** | | **Kansas (KS)** | |
| --- | --- | --- | --- | --- | --- | --- |
| **Generation** | **Selected^a^** | **Control** | **Selected^a^** | **Control** | **Selected** | **Control** |
| 1 |  | Isoline |  | Isoline | Bt | Isoline |
| 2 |  | Isoline |  | Isoline | Isoline | Isoline |
| 3 |  | Isoline |  | Isoline | Bt | Isoline |
| 4 |  | Isoline |  | Isoline | Isoline | Isoline |
| 5 |  | Isoline | Bt | Isoline | Bt* | Isoline* |
| 6 |  | Isoline | Bt | Isoline | Isoline | Isoline† |
| 7 | Bt | Isoline | Isoline | Isoline* | Bt† | Isoline |
| 8 | Isoline | Isoline* | Bt* | Isoline | Bt | Isoline |
| 9 | Bt | Isoline | Bt | Isoline | Isoline | Isoline |
| 10 | Isoline | Isoline | Bt**† | Isoline | Bt | Isoline |
| 11 | Bt* | Isoline**† | Isoline | Isoline**† | Bt** | Isoline |
| 12 | Bt**† | Isoline | Bt | Isoline | Isoline | Isoline** |
| 13 | Isoline | Isoline | Isoline | Isoline† |  | Isoline |

^a^ The MN and WI selected colonies collapsed after three and two generations,

respectively, of initial rearing on Bt corn. We began selection again using a

subset of the control colony for each of these origins, after rearing on isoline

corn for six generations for MN and four generations for WI.

* Greenhouse test on Bt corn and isoline corn

** Field test on Bt corn and isoline corn

† Fitness cost tests on isoline corn only for larval performance in the greenhouse

and adult performance in the laboratory
